# Supplementary material for: AudBility: Effectiveness of an online central auditory processing screening program
Source: PLoS One. 2021 Aug 30;16(8):e0256593. doi: 10.1371/journal.pone.0256593 (PMC8405004; doi:10.1371/journal.pone.0256593)
Supplement: Attachment — Submitted filename: Answer to the reviewers PLOSONE.docx [file pone.0256593.s001.docx]

**Answer to the reviewers**

First and foremost, we would like to thank you for your comments and suggestions. They were taken into account and have been implemented in our manuscript, which made it more appropriate and more interesting for publication. Therefore, please accept this new revised version for analysis.

Reviewer #1: *In Methods the authors state something about a "second stage." But I do not see any further discussion of a second stage, so what does that mean?*

**Answer:** This means that the study was applied in two stages, in two different locations. In the first stage, the screening was carried out at a school. In the second stage, students were invited to attend Laboratory of Institution for formal assessment of central auditory processing within a 72-hour period. In this second stage, we rely on the voluntary displacement of children and their families.

*Line 32 The authors use the term "module" but I think that is the wrong word and they mean to discuss "the age range of subjects is....."*

**Answer:** Thanks for the correction. Initially we thought of a module because AudBility was divided with tasks designed for children between 6 and 8 years old and above 9 years old. We made the correction and highlighted it in the text.

*Line 54 The authors discuss "better than the eight year olds." Better is a relative term, better than what? I think the authors need to reword that sentence.*

**Answer:** Thanks for the correction. The term better is together with performance, results or effectiveness.

*Line 172 or so the word "WAS" appears, I think that is an error*

**Answer:** Thanks for the correction. This word has been deleted.  *Line 183 Add the word and "...and better..."*

**Answer:** Thanks for the correction. The word and has been added. *Line 194 The authors need to use larger fonts for the figure legends. The font they use is difficult to read. Perhaps the journal redactor would have done that - but it needs to be changed.*

**Answer:** Thanks for the correction. The font has been altered.

*Line 253 I made a comment but cannot read my own handwriting. That's embarrassing. But the comment has to do with transition to adults 29?*

**Answer:** The cited literature [29] has already reported that “Children of 5 yr of age did not perform significantly different from adults on the sound localization task”. Therefore, it is not surprising that from the age of 6 the performance became easy. *Line 325 The text should read something like: "...with better effectiveness than...eight year olds."*

**Answer:** Thanks for the correction.

Reviewer #2: *Thank you for sending me this manuscript. Firstly, The seccion of the limitation must be add. Every single publication have the limitations so it is crucial to add this secction. Then the checking of the manuscript will be possible.*

**Answer:** We appreciate and agree with your suggestion. The limitations section was added. Please check in the text.
