# Supplementary material for: AudBility: Effectiveness of an online central auditory processing screening program
Source: PLoS One. 2021 Aug 30;16(8):e0256593. doi: 10.1371/journal.pone.0256593 (PMC8405004; doi:10.1371/journal.pone.0256593)
Supplement: Attachment — Submitted filename: Answer to the reviewers minor revision.docx [file pone.0256593.s002.docx]

**Answer to the reviewers**

First and foremost, we would like to thank you for new comments and suggestions. They were taken into account and have been implemented in our manuscript. Therefore, please accept again this version with minor revision.

**Reviewer #1**: *275 The value of accuracy in the auditory closure (AF) screening task could (note that AF is wrong. Line 275 should read, “….. in the auditory closure (AC) screening….*

**Answer:** There was a typo. We appreciate the correction.

*In the summary spell out AD, FG and DD. The abbreviations do not work here, since some readers will go to the summery to decide whether to read the entire article. For example: I think that AD that is shown above as copied from the manuscript is not correct - that the line should read auditory closure as AD and not AD 275. The value of accuracy in the auditory closure (AF) screening task accuracy values were AC (76.9%); FG (61.6%); DD 78.8% dichotic digit screening task -binaural integration (DD) figure-ground screening (FG) task.*

**Answer:** Abbreviations have been replaced by task names.

*Revise the line 354 where the English on limitations is not quite correct from:
354 The study has limitations in relation the was not application of formal tests to assess language and cognitive aspects. In addition, peripheral cochlear screening was not performed.*

**Answer:** The English has been revised.

**Reviewer #2:** *Thank you for giving me the opportunity to revise the manuscript “effectiveness of an online central auditory processing screening program” for publication in the Journal. I appreciate the time and effort that authors dedicated to prepare and conduct this study. Firstly, It would be great if linguistic proofreading will be done. My comments: L54: change eight into 8
Could you add table were according to the PICO classification (Population with inclusion and exclusion criteria, I - intervention, C - comparator, o- outcome) you will explain each point? The limitations of this study should be extended and the risk of this limitation should be add at the discussion.*

**Answer:** We appreciate the comments. Changes were implemented in the manuscript. The linguistic proofreading was done; has been changed for eight. The PICO classification was added, and limitations has been extended.
